# Integrated Metabolomic and Network Analysis to Explore the Potential Mechanism of Three Chemical Elicitors in Rapamycin Overproduction

**DOI:** 10.3390/microorganisms10112205

**Published:** 2022-11-08

**Authors:** Dandan Zhang, Jinyu Chen, Zihui Wang, Cheng Wang

**Affiliations:** College of Forestry, Northwest A&F University, Yangling, Xianyang 712100, China

**Keywords:** rapamycin, chemical elicitors, metabolomics, metabolic network, *S. hygroscopicus*

## Abstract

Rapamycin is a polyketide macrocyclic antibiotic with exceptional pharmacological potential. To explore the potential mechanism of rapamycin overproduction, the intracellular metabolic differences of three chemical elicitor treatments were first investigated by combining them with dynamic metabolomics and network analysis. The metabolic response characteristics of each chemical elicitor treatment were identified by a weighted gene co-expression network analysis (WGCNA) model. According to the analysis of the identified metabolic modules, the changes in the cell membrane permeability might play a key role in rapamycin overproduction for dimethyl sulfoxide (DMSO) treatment. The enhancement of the starter unit of 4,5-dihydroxycyclohex-1-ene carboxylic acid (DHCHC) and the nicotinamide adenine dinucleotide phosphate (NADPH) availability were the main functions in the LaCl_3_ treatment. However, for sodium butyrate (SB), the improvement of the methylmalonyl-CoA and NADPH availability was a potential reason for the rapamycin overproduction. Further, the responsive metabolic pathways after chemical elicitor treatments were selected to predict the potential key limiting steps in rapamycin accumulation using a genome-scale metabolic network model (GSMM). Based on the prediction results, the targets within the reinforcement of the DHCHC and NADPH supply were selected to verify their effects on rapamycin production. The highest rapamycin yield improved 1.62 fold in the HT-aroA/zwf2 strain compared to the control.

## 1. Introduction

Rapamycin is a 31-membered polyketide macrocyclic antibiotic, which can be produced by several actinomycetes (e.g., *Streptomyces hygroscopicus*, *Streptomyces iranensis*, *Actinoplanes* sp. N902-109) [1]. Since the discovery of rapamycin from Easter Island in 1975 [2], it has attracted interest from several research groups because of its exceptional pharmacological potential, such as its immunosuppressive [2], antifungal [2], antitumor [3], neuroprotective/neuroregenerative [4], and lifespan extension activities [5]. With its increasing application, the massive demand of rapamycin in the global antibiotic market has also posed a unique challenge for improving rapamycin yield [6].

Nowadays, the details of rapamycin biosynthesis have been clarified [1], and it starts from a 4,5-dihydroxycyclohex-1-ene carboxylic acid (DHCHC) unit from the shikimic acid branching pathway. Then, a series of condensation reactions from acetate and propionate building blocks take place to form a nascent polyketide chain through the polyketide pathway. The linear polyketide chain is subsequently condensed with lysine-derived pipecolate, and the macrolide ring is further cyclized and modified to form rapamycin. During the above process, there are close connections between the primary and secondary metabolism in the rapamycin production, both of which involve precursor biosynthesis and nutrient regulation. Therefore, how to improve rapamycin yield effectively has aroused great interest from researchers and pharmaceutical companies. In previous reports, many kinds of strategies have been applied to improve rapamycin yield, including strain mutagenesis [6,7,8,9], bioprocess optimization [10,11], strengthening the precursor supply [12,13,14], and engineering the regulatory genes of the rapamycin biosynthetic cluster [15]. While a significant improvement in rapamycin yields has been observed in the above-mentioned literatures, knowledge of the multiple regulatory cascades and networks is still finite in rapamycin biosynthesis (except for the biosynthetic gene clusters of rapamycin). 

In fact, some secondary metabolites are likely only synthesized under specific conditions. To explore the potential mechanism of secondary metabolite synthesis, therefore, some stress responses were applied to trigger the expression of secondary metabolic genes (e.g., heat and ethanol shock, chemical elicitors) [16]. Some cryptic pathways or genes were activated, which might act as the key limiting factors in improving the secondary metabolite yield. For example, our group found that various chemical elicitors (e.g., dimethyl sulfoxide (DMSO), sodium butyrate, and rare earth elements) showed stronger promoting effects on tacrolimus accumulation (another macrolide antibiotic) in *Streptomyces tsukubaensis* [17]. Similar effects had also been found in ascomycin production by the exogenous feeding strategies of DMSO [18], and the final ascomycin yield reached 1258 mg/L using the combinatorial engineering approach identified in DMSO treatment. Therefore, the exploration and application of the chemical elicitors will provide an effective strategy for a comprehensive understanding of the potential metabolic regulation of rapamycin biosynthesis. 

In recent years, metabolomics have acted as an effective strategy to directly discover the metabolic differences caused by environmental disturbances, such as carbon repression [19], salt stress [20], and primary metabolic pathway disturbances [21]. Combined with multivariate statistics analysis methods (e.g., principal component analysis (PCA), orthogonal partial least squares discriminant analysis (OPLS-DA), hierarchical clustering (HCL), and weighted gene co-expression network analysis (WGCNA)), notably, metabolomics is widely applied to deeply understand the function of metabolites in a given metabolic pathway or their relationship to other metabolites in the entire intracellular metabolic network. For example, the systematic strategies of exogenous feeding reinforcement and genetic engineering were proposed to improve the secondary metabolite yields in different *Streptomyces* species, including *S. hygroscopicus* [13,18,22], *S. tsukubaensis* [21], *S. coelicolor* [20], *S. lydicus* [23,24], and so on. These reports also demonstrated that metabolomics is a suitable tool in exploring and understanding the changes in the intracellular metabolism under exogenous or endogenous disturbances. 

In this work, three kinds of chemical elicitors (i.e., DMSO, LaCl_3_, and sodium butyrate (SB)) were selected to investigate their effects on rapamycin accumulation. Subsequently, the metabolomics associated with different multivariate statistical analyses were applied to analyze the dynamic characteristics of the intracellular metabolism, including the key biomarker, and the potential key metabolic pathways and nodes. The potential key limiting steps were further identified in the above key metabolic pathways based on a genome-scale metabolic network model (GSMM) of *S. hygroscopicus* constructed by our group before. After the corresponding gene manipulation, these identified targets were verified in experiments. This strategy is not only helpful for a better understanding of rapamycin biosynthesis and its potential intracellular metabolic regulation but also provides valuable guidance on other secondary metabolite overproduction.

## 2. Materials and Methods

### 2.1. Strains and Cultivation

The parent strain of *S. hygroscopicus* HA12 was derived from the wild-type strain *S. hygroscopicus* ATCC 29253 after several rounds of ultraviolet mutagenesis. The spores and seed medium of *S. hygroscopicus* HA12 and its engineered strains were consistent with the previous reported methods by our group before [12,13]. The fermentation culture was performed in a 500 mL Erlenmeyer flask with the following medium: 30.0 g/L glucose, 30.0 g/L soluble starch, 20.0 g/L soybean meal, 2 g/L (NH_4_)_2_SO_4_, and 1 g/L K_2_HPO_4_ and adjusted to pH 7.0 before autoclaving. After 10% (*v*/*v*) seed culture was added into the fermentation medium, the batch fermentation was performed at 28 °C, 220 rpm for 7 days. Except for DMSO, the rest of the chemical elicitors were first dissolved by distilled water. Then, all of the chemical elicitor solutions were filtered with sterile filtration (0.22 μm) before being used. All plasmids and strains used in the gene-engineered manipulation are described in Table S1. Luria-Bertani medium was used in *E. coli* cultivation.

### 2.2. Chemicals

DMSO, LaCl_3_, and sodium butyrate at high purity were of analytical grade and purchased from Tianjin Guangfu Technology Co., Ltd. (Tianjin, China). All of the antibiotics (e.g., kanamycin, chloramphenicol, ampicillin) used in gene manipulation were purchased from Sangon Biotech (Shanghai) Co., Ltd. (Shanghai, China). The standard of rapamycin was purchased from Sigma-Aldrich (St. Louis, MO, USA). All of the restriction endonucleases were purchased from Fermentas Inc. (Waltham, MA, USA), and the rest of the enzymes were purchased from TransGen Biotech Co., Ltd. (Beijing, China). All of the chemicals (chromatographic purity) used in metabolomics analysis were purchased from Sigma-Aldrich. Except for the above-mentioned chemical reagents, the rest of the chemicals were of analytical grade and purchased from Tianjin Bodi Chemicals Co., Ltd. (Tianjin, China) and Chengdu Kelon Chemical Reagent Co., Ltd. (Chengdu, China).

### 2.3. Manipulation of the Genes Deletion or Overexpression

All *Streptomyces* DNA manipulations and transformations were performed using the standard protocols [25]. All of the used primers are listed in Table S2. For single gene overexpression, the genes of *aro*A, *tkt*B, and *zwf*2 were first amplified by PCR using primers of *aro*A-F^1^/*aro*A-R^1^, *tkt*B-F^1^/*tkt*B-R^1^, and *zwf*2-F^1^/*zwf*2-R^1^. The above PCR products were digested by NdeI-XbaI and cloned into pIB139 to generate pAROA, pTKTB, and pZWF2, respectively. In the construction of the vector pIB139-*aro*A/*tkt*B (pAT), the genes of *aro*A and *tkt*B were amplified using the primer pairs of *aro*A-F^2^/*aro*A-R^2^ and *tkt*B-F^2^/*tkt*B-R^2^, respectively. The *aro*A PCR product was first digested by NdeI-HindIII and cloned into pUC18 to generate pUC18-A. The plasmid of pUC18-A was further cut by HindIII-XbaI and linked with the *tkt*B PCR product (also digested by HindIII-XbaI) to form pUC18-AT. Finally, the plasmid pUC18-AT was excised with NdeI-XbaI and cloned the two genes into pIB139 to generate pAT. Similarly, the plasmids of pUC18-AZ and pAZ were also constructed using the above methods. All of the overexpression plasmids (i.e., pAROA, pTKTB, pZWF2, and pAT) were introduced into the parental strain using the previous described methods [17]. All of the positive mutants were verified using PCR amplification and DNA sequencing.

To delete the gene *gdh*A, the upstream flanking fragment of *gdh*A was first amplified by PCR using primers of *gdh*A-LF/*gdh*A-LR, and its downstream flanking fragment was further amplified using primers of *gdh*A-RF/*gdh*A-RR. Then, the above fused fragments were ligated into HindIII-EcoRI-digested pKC1139 to obtain the *gdh*A deletion vector p△GCDH. Transformation of p△GCDH into the parental strain was carried out as described before [17]. The putative *gdh*A deletion mutant was verified by the same methods used in the above single gene overexpression.

For the combined gene overexpression and knockout manipulation, the vector pAROA was transferred into the HT-∆gdhA strain to generate HT-∆gdhA-aroA strain, using the reported methods [17]. The positive mutant was verified by the same methods used in the above single gene overexpression.

### 2.4. Detection of the Basic Fermentation Parameters

Approximately 15 mL of fermentation broth was used at each sample point for the following detection. Biomass was detected by the method of dry cell weight [17]. Rapamycin was measured using the described method used by our group before [12,13]. The other basic fermentation parameters were determined by the reported methods [26], including the residual total glucose, inorganic nitrogen (NH_4_^+^ form), and phosphorus (PO_4_^3+^ form).

### 2.5. Intercellular Metabolites Analysis

To explore the dynamic changes of intercellular metabolism under different chemical elicitor treatments, the fermentation samples treated with DMSO, LaCl_3_, and sodium butyrate (SB) at different time points were collected for intercellular metabolite analysis, respectively. The corresponding methods in the extraction and detection of intracellular metabolites were all consisted with the reported methods by our group [17], including GC-MS (Table S3, 82 metabolites) and ultraperformance (UP)LC-MS/MS (Tables S3 and S4, 10 sugar phosphates) analyses.

### 2.6. Screening of the Potential Key Genes by GSMM Model

The previous constructed GSMM model of *S. hygroscopicus* by our group before was used to predict the potential targets of enhancing rapamycin production in the identified metabolic pathways of metabolomic analysis [12]. Combined with minimization of metabolic adjustment (MOMA) into flux balance analysis (FBA) algorithm, the simulated manipulation of the gene overexpression and knockout was performed by regulating the metabolic flux of each metabolic reaction in GSMM model [12,27]. During the identification of the potential targets, *f_PH_* (the ratio of weighted and dimensionless specific growth rate and specific rapamycin production rate) values of each target were compared based on the reported methods [27].

### 2.7. Data Processing and Multivariate Statistical Analysis

According to the previously reported method [17], all of the metabolomic datasets were simultaneously normalized before the multivariate statistical analysis. PCA and partial least squares discriminant analyses (PLS-DA) were performed using SIMCA-P package (Version 11.5; Umetrics, Umea, Sweden). The scores of variable importance of projection (VIP) were calculated for each metabolite after PLS-DA analysis. R software was performed on WGCNA analysis based on the reported methods [28]. A web-based metabolomics pathway analysis (MetPA) platform (http://www.metaboanalyst.ca, accessed on 10 January 2021) was used for the pathway enrichment analysis of the metabolites identified by WGCNA [29]. Significant difference between the samples was determined by Duncan’s test (SPSS 20, IBM Corporation, Armonk, NY, USA). Difference at *p* < 0.05 was considered as significant. Four biological replicates were performed in the above detection.

## 3. Results

### 3.1. Effects of Different Chemical Elicitors on Rapamycin Accumulation

**Figure 1:**
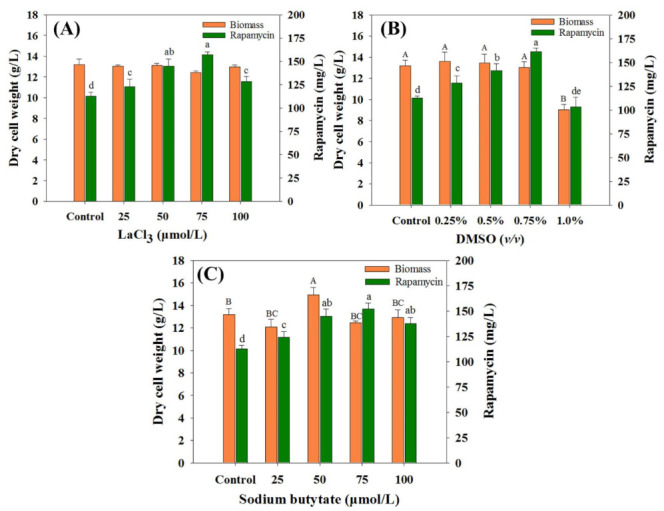
Effects of chemical elicitors on biomass and rapamycin yields. (**A**) Dimethyl sulfoxide (DMSO), (**B**) LaCl_3_, (**C**) sodium butyrate (SB). The final biomass and rapamycin contents were determined after seven days of fermentation. The significant difference levels (*p* < 0.05) of biomass and rapamycin contents among the different concentrations of each chemical elicitor were marked with uppercase (biomass, e.g., A, B, C) and lowercase (rapamycin, e.g., a, b, c) letters, respectively.

As an effective method in enhancing secondary metabolite production, chemical elicitors play important roles in the activation of cryptic pathways or large and complex regulatory networks [16]. Therefore, to comprehensively evaluate the effects of the selected chemical elicitors on rapamycin yields, the optimal concentration of each chemical elicitor was first investigated. As shown in Figure 1, the final rapamycin production was enhanced to varying degrees within most of the chemical elicitor treatments (except for DMSO at 1.0%) compared with the control (112.59 mg/mL). For each kind of chemical elicitor, the highest rapamycin yields were found in DMSO (0.75% (*v*/*v*), 161.16 mg/mL), followed by LaCl_3_ (75 μmol/L, 157.03 mg/mL) and SB (75 μmol/L, 151.80 mg/mL). Interestingly, no obvious differences were observed in the biomass contents between the control and the treatment groups with the optimal concentration conditions. This indicates that the intracellular metabolism might be slightly disturbed under the current feeding strategy of all of the selected chemical elicitors.

**Figure 2:**
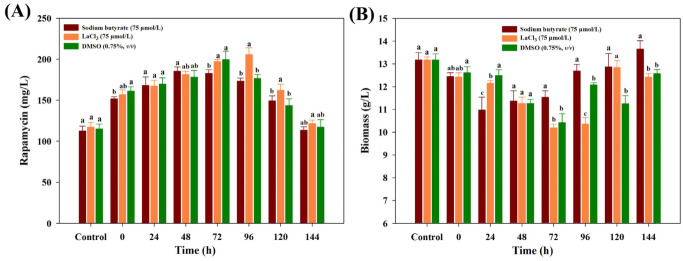
Effects of different feeding time points on the final rapamycin production (**A**) and biomass contents (**B**) under the three kinds of chemical elicitor treatments. Three biological replicates were used to perform multivariate analysis of each treatment. The significant differences (*p* < 0.05) of rapamycin yields were marked with the lowercase letters among the different strains.

To further ascertain the best feeding time, each chemical elicitor with the optimal concentration was added into the medium under different fermentation times. While the similar tendency of rapamycin yields was also observed in all treatments, obvious differences were found in each chemical elicitor treatment. Among all of the treatments, the highest yields of rapamycin were found in LaCl_3_ at 96 h (205.67 mg/mL), followed by DMSO (72 h, 199.56 mg/mL) and SB (48 h, 185.43 mg/mL), respectively (Figure 2A). At the highest rapamycin yields, the final biomass contents of each chemical elicitor treatment significantly decreased in all treatments (Figure 2B). A possible reason is that the intracellular metabolism might be significantly disturbed after feeding the chemical elicitor treatments, and more metabolic fluxes flowed to the biosynthetic pathways of rapamycin. However, what is actual going on inside the intracellular metabolism is still unknown for each chemical elicitor treatment. Therefore, the dynamic changes of intracellular metabolites will be further analyzed.

### 3.2. Metabolomic Profiles of Different Chemical Elicitor Treatments

To facilitate the comparison of the intracellular metabolic differences, the above three chemical elicitors were simultaneously added into the fermentation medium at 72 h of the batch fermentation. At this feeding time point, in fact, there were no obvious differences in the rapamycin yields in the SB and LaCl_3_ treatments compared to their optimal feeding times (Figure 2A). Combined with the datasets of GC-MS and LC-MS/MS, therefore, the dynamic changes of the intracellular metabolites were further explored. According to the above strategies, three different sampling times (i.e., 12 h, 24 h, and 48 h after the feeding of the three chemical elicitors) were further selected to investigate the intracellular metabolic characteristics in each chemical elicitor treatment. Finally, there was a total of 93 intracellular metabolites, including 83 metabolites by GC-MS analysis and 10 sugar phosphates by LC-MS/MS analysis.

**Figure 3:**
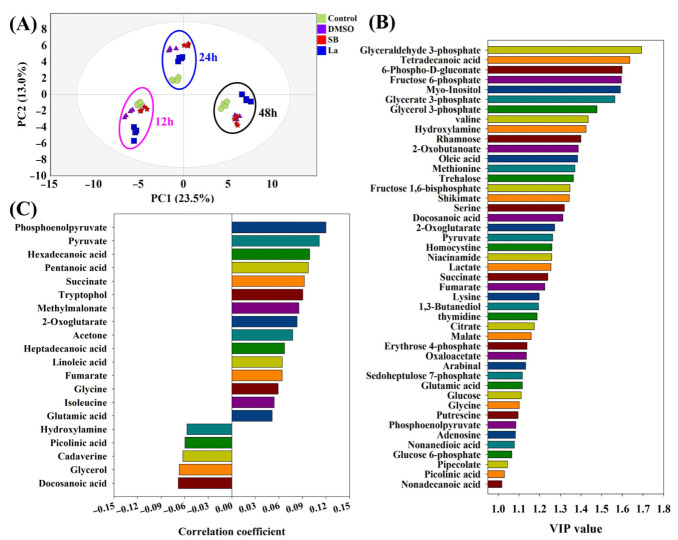
PCA and PLS-DA analyses of intracellular metabolites under different chemical elicitor treatments. Compared with the control, DMSO, SB, and LaCl_3_ were added into the medium at 72 h of the batch fermentation, respectively. After an additional 12 h, 24 h, and 48 h of culture, intracellular metabolites of each sampling time point were analyzed by different multivariate statistical analysis methods, respectively. (**A**) PCA-derived score plots; (**B**) VIP score plot derived from PLS-DA analysis; (**C**) the metabolites correlated with rapamycin using PLS-DA.

According to the results of the PCA analysis (Figure 3A), the obvious characteristics observed were as follows: (i) all of the samples within the same time points could be clustered together and separated with other time point samples; (ii) the distinct separation of each sample was only found at 24 h after the feedings of the chemical elicitors. The samples under LaCl_3_ treatment exhibited the longest distances compared to the rest of the groups at all of the sample time points. The longer distances in the PCA score plots might also indicate that larger differences existed in the intracellular metabolism during the LaCl_3_ treatment compared to other treatment groups. Therefore, to search the differential metabolites, PLS-DA was further performed to evaluate the contribution of each metabolite on the differences in all of the samples and the rapamycin accumulation, respectively. Furthermore, 45 metabolites with the higher VIP values (VIP score > 1.0) generated by the PLS-DA were identified, which could be recognized as the potential biomarkers under chemical elicitor treatments (Figure 3B). Among these identified metabolites, the first six metabolites within the higher VIP values (>1.5) were mostly derived from glycolysis (e.g., glyceraldehyde 3-phosphate, fructose 6-phosphate, glycerate 3-phosphate) and the pentose phosphate (PP) pathway (e.g., 6-phospho-D-gluconate). As the most important carbon metabolism, the identification of these metabolic pathways suggests the carbon fluxes of the intracellular metabolism might be disturbed after chemical elicitor treatments. Moreover, using rapamycin yields as Y values, 20 metabolites exhibited a higher association with rapamycin accumulation based on their correlation coefficient (Figure 3C). Therefore, phosphoenolpyruvate (PEP) and pyruvate showed the strongest positive correlation with rapamycin biosynthesis in all of the identified metabolites. In the rapamycin biosynthetic pathways, several precursors were derived from these two metabolic nodes. For example, the starter unit of rapamycin (i.e., DHCHC) was synthesized by the shikimate pathway, which originated from the reaction of PEP and erythrose 4-phosphate. Meanwhile, as the important starting point of CoA compounds synthesis, pyruvate could be transformed into acetyl-CoA and propionyl-CoA. Both of them can further form the direct precursors of rapamycin (i.e., malonyl-CoA and methylmalonyl-CoA). Additionally, these two metabolic nodes were also directly connected with several kinds of amino acid biosynthesis and TCA cycles. Therefore, the regulation of the metabolic fluxes around these two key metabolic nodes was successfully applied in the overproduction of several polyketide macrocyclic antibiotics, including tacrolimus [21,27] and ascomycin [18,22]. Interestingly, two kinds of fatty acids (i.e., hexadecenoic acid and pentanoic acid) also showed a stronger positive correlation with rapamycin accumulation. In the previous reports, it was confirmed that the activated fatty acid metabolism could supply higher levels of malonyl-CoA, methylmalonyl-CoA, and acetyl-CoA in tacrolimus fermentation [30]. Additionally, other metabolites that related to the precursor biosynthesis of rapamycin were also observed using PLS-DA analysis, such as succinate and methylmalonate. The above information indicated that the precursor synthetic pathways of rapamycin had been effectively activated after the chemical elicitor treatments. However, the specific differences in the intracellular metabolism of each chemical elicitor were still unknown. Therefore, the further analysis will be investigated in the following work.

### 3.3. Module Characteristics of the Intracellular Metabolism of Each Chemical Elicitor Treatment

**Figure 4:**
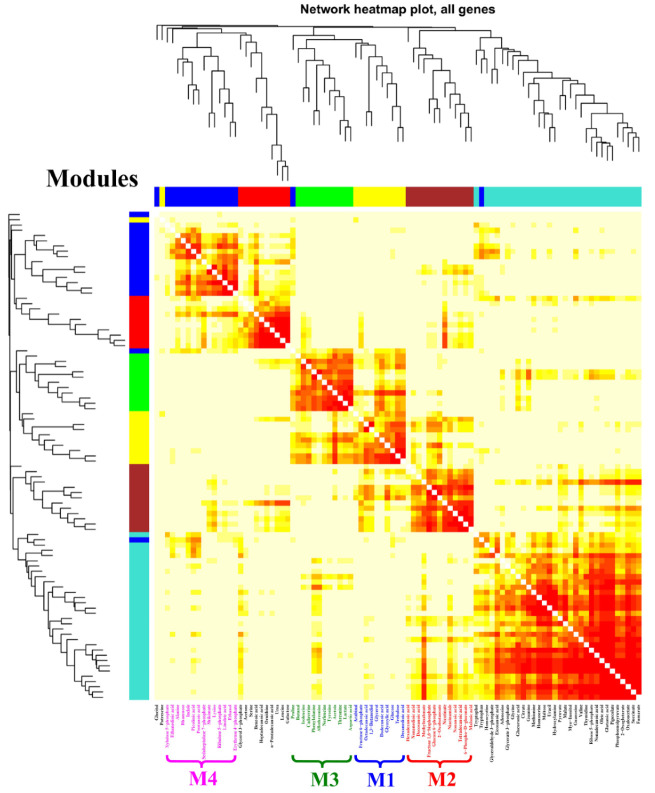
WGCNA analysis of metabolic profiles in *S. hygroscopicus* under three kinds of chemical elicitor treatments. Three chemical elicitors were simultaneously added into the medium at 72 h of the fermentation (i.e., DMSO, LaCl_3_, sodium butyrate (SB)). The metabolomics dataset was derived from the samples collected at 24 h after feedings of the chemical elicitors. The metabolite information of each identified module is also listed in Table 1 and Figure 5.

According to the results of the PCA score plots (Figure 3A), it was clear that the significant separation could only be observed at 24 h after feeding the chemical elicitors. Therefore, a WGNCA using the metabolomic datasets at this time sampling point was constructed to explore the metabolic response characteristics for each chemical elicitor treatment. Using a cut-off of correlation coefficients *r* > 0.65 and a statistical significance of *p* value < 0.01, four distinct metabolic modules were identified and exhibited a highly positive association with DMSO (M1), SB (M2 and M3), and LaCl_3_ (M4) treatments, respectively (Figure 4 and Table 1).

**Figure 5:**
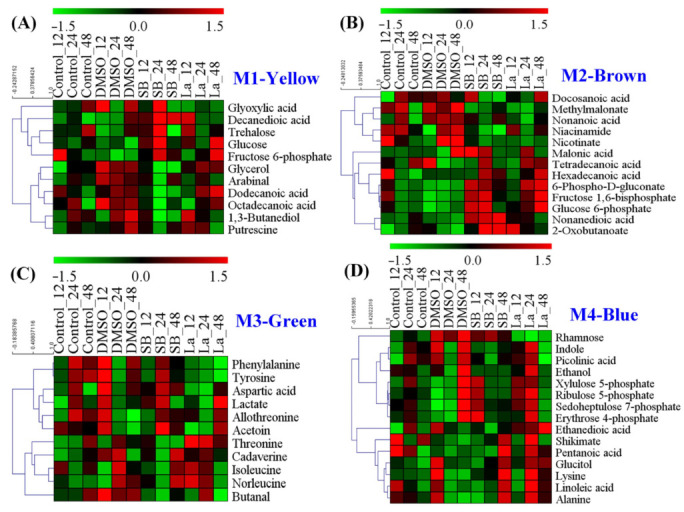
Heat map analysis of the metabolites identified in each distinct metabolic module of WGCNA analysis. (**A**) The metabolic module (M1) showed a highly positive association with DMSO; (**B**,**C**) the metabolic modules (M3 and M4) showed a highly positive association with sodium butyrate (SB); (**D**) the metabolic module (M4) showed a highly positive association with LaCl_3_.

For the DMSO treatment, a total of 11 characteristic metabolites were observed in Module 1 (Figure 5A). Using the *p* value < 0.05 and metabolite number ≥ 3 as screening parameters, no metabolic pathways were enriched by the MetPA analysis. In the previous literature, numerous studies confirmed that DMSO can effectively enhance several antibiotic production, such as ascomycin [18], tacrolimus [17], tylosin [31], thiostrepton, and tetracenomycin [32]. However, the specific mechanism of DMSO on secondary metabolite overproduction is still unknown. Under normal conditions, DMSO is usually known as the enhancer of membrane permeation in the pharmaceutical industry [33], which helps active molecules cross the cell membranes by decreasing the thickness of the lipid bilayer [34]. As a kind of biomacromolecule, the excessive accumulation of rapamycin in the intracellular would be harmful to the strain cellular. Therefore, an increase in the cell membrane permeability might be suitable for the transport of rapamycin from the intracellular to extracellular. Meanwhile, among all of the identified metabolites, several metabolites related to fatty acid metabolism were observed, such as dodecanoic acid, decanedioic acid, octadecanoic acid, and glycerol. In fact, fatty acids are the important constituent part of the cell membrane, which can change the fluidity of the cell membrane by regulating the ratio of saturated and unsaturated fatty acids. Additionally, two kinds of stress response factors (i.e., trehalose and putrescine) were also found in the DMSO treatment. Furthermore, it was reported that putrescine played important roles in the regulation of proteins and the fatty acids of cell membranes under salt stress conditions [35]. Taking all of the above into consideration, we speculate that an increase in cell membrane permeability might play a key role in rapamycin overproduction for DMSO treatments.

Different to DMSO, two distinct metabolic modules were identified in the SB treatment, including Module 2 (13 metabolites) and Module 3 (11 metabolites) (Figure 5B,C). Taken these metabolites together, four metabolic pathways were successfully enriched by MetPA analysis, including aminoacyl-tRNA biosynthesis, nicotinate and nicotinamide metabolism, glycine, serine and threonine metabolism, valine, leucine, and isoleucine biosynthesis (Figure S1A). In previous reports, amino acid metabolism was also confirmed to be crucial in rapamycin accumulation [8]. For example, valine, leucine, and isoleucine are all precursors of methylmalonyl-CoA in rapamycin biosynthesis. Meanwhile, it was reported that nicotinic acid and nicotinamide acted as growth stimulators to improve tacrolimus production by the stimulation of NAD/NADP biosynthesis in *S. tsukubaensis* [36]. In fact, as a kind of histone deacetylase (HDACs) inhibitor, SB can change the chromatin structure by antagonizing the acetylation of histones and activate the gene expression within the biosynthetic clusters of natural products [16]. Therefore, an improvement in the direct precursor availability (e.g., methylmalonyl-CoA and NADPH) might be a potential reason for the rapamycin overproduction.

For the LaCl_3_ treatment, only one metabolic module (Module 4) was identified, which contained 17 characteristic metabolites. Using MetPA analysis, three metabolic pathways were successfully enriched, including phenylalanine, tyrosine and tryptophan biosynthesis, PP pathway, and aminoacyl-tRNA biosynthesis (Figure S1B). Interestingly, in addition to aminoacyl-tRNA biosynthesis, the rest of the two metabolic pathways played important roles in the key precursors of DHCHC and NADPH during rapamycin biosynthesis. For example, the activation of the PP pathway cannot only provide more reducing power via NADPH but also promote the synthesis of aromatic amino acids (i.e., phenylalanine, tyrosine, and tryptophan) by enhancing the level of erythrose 4-phosphate in the shikimate pathway. As shown in Figure 5D, the relative level of most identified metabolites (except for rhamnose) showed the higher abundances at the selected sampling time point. In fact, the shikimate pathway may not only be the only synthetic pathway of the aromatic amino acids but also the start point of rapamycin biosynthesis. Specifically, DHCHC was synthesized from the key metabolic branch of chorismate, which controlled the transition of the primary metabolism (i.e., phenylalanine, tyrosine, and tryptophan biosynthesis) into the secondary metabolism (i.e., DHCHC). Therefore, the identification of these metabolic pathways suggest that the enhancement of the starter unit of DHCHC and NADPH played a key role in the LaCl_3_ treatment.

### 3.4. Identification of the Key Limiting Steps using GSMM Analysis

To ascertain the key limiting steps in the above enriched metabolic pathways, the GSMM model constructed by our group before was further performed to predict the potential targets. Under the current fermentation conditions, the parts of the basic fermentation parameters were first determined and set as the constrain conditions of the simulation (Figure S2). For example, the upper bound of the exchange reactions for the specific uptake rates of glucose, inorganic nitrogen (NH_4_^+^ form), and inorganic phosphorus (PO_4_^3+^ form) were set to 1.08, 0.132, and 0.0074 mmol/(g DCW•h), respectively. The experimental specific production rate of rapamycin (0.021 μmol/(g DCW•h)) was set to the lower bound of the rapamycin exchange reaction. Using these experimental constrains, the specific growth rate was predicted to be 0.0565 h^−1^, which was close to the experimental data (0.061 h^−1^).

**Figure 6:**
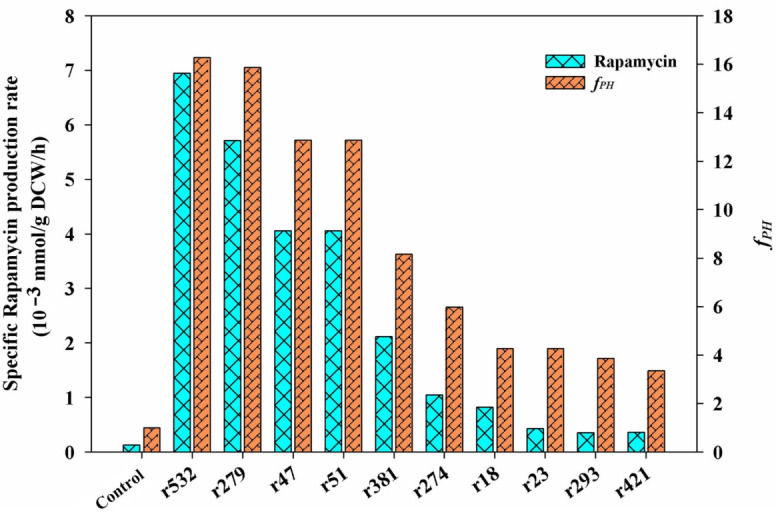
The predicted key limiting targets using GSMM model. According to the results of the metabolic characteristic analysis of each chemical elicitor treatment, all of the candidate targets were selected from the enriched metabolic pathways, including the central carbon metabolism, pyruvate metabolism, and the metabolism of several amino acids (i.e., phenylalanine, tyrosine and tryptophan biosynthesis, alanine, aspartate and glutamate metabolism, glycine, serine and threonine metabolism, valine, leucine, and isoleucine biosynthesis). Only the first ten targets are listed, and the detailed information of each target can also be seen in Table 2.

Among the selected metabolic pathways (i.e., the central carbon metabolism, pyruvate metabolism, and the metabolism of several amino acids), the first 10 potential targets with a higher *f_PH_* value were listed (Figure 6), and their detailed reaction information is also described in Table 2. Notably, among all of the listed targets, the first four targets (i.e., r532, r279, r47, and r51) showed the same features in the potential mechanism of rapamycin overproduction. All of the simulated results of the gene manipulation showed an increase in the NADPH level and the metabolic flux of the shikimate pathway. For example, as the first step of the shikimate pathway, the overexpression of the target r532 (*aro*A, 2-dehydro-3-deoxyphosphoheptonate aldolase) would be beneficial for the synthesis of the start unit of rapamycin (i.e., DHCHC). In addition to NADPH, the intermediate metabolite (i.e, erythrose-4-phosphate) derived from the PP pathway is the direct precursor of the shikimate pathway. Therefore, the overexpression targets of r47 and r51 would achieve the same aims in improving the supply of the DHCHC and NADPH levels. Similarly, the knockout of the target r75 (*gdh*A, glutamate dehydrogenase) could directly increase the level of NADPH availability, which was confirmed in other antibiotics overproduction (e.g., tacrolimus) [27].

For the rest of the predicted targets, the potential reasons for the higher rapamycin production can also be divided into two kinds of patterns. The first one is the regulation of the carbon flux distribution in the key metabolic nodes of PEP and pyruvate. For example, the inactivation of the targets r18 and r23 can decrease the branch metabolism of the above two metabolites. However, it would be beneficial for the accumulation of the intermediate precursors of rapamycin synthesized from these two metabolites (e.g., DHCHC, methylmalonyl-CoA, acetyl-CoA). The other is to increase the level of pipecolate by enhancing the metabolic flux of aspartate (or lysine) biosynthesis (e.g., r274, r293, r421) directly or by blocking the branched metabolic pathway of the lysine degradation (e.g., r381). As the key precursor of rapamycin biosynthesis, the transformation of lysine is the only source of pipecolate biosynthesis. Therefore, the enhancement of aspartate metabolism (especially for lysine and pipecolate biosynthesis) could improve the yields of rapamycin.

### 3.5. Modification of the Potential Targets to Improve Rapamycin Production

**Figure 7:**
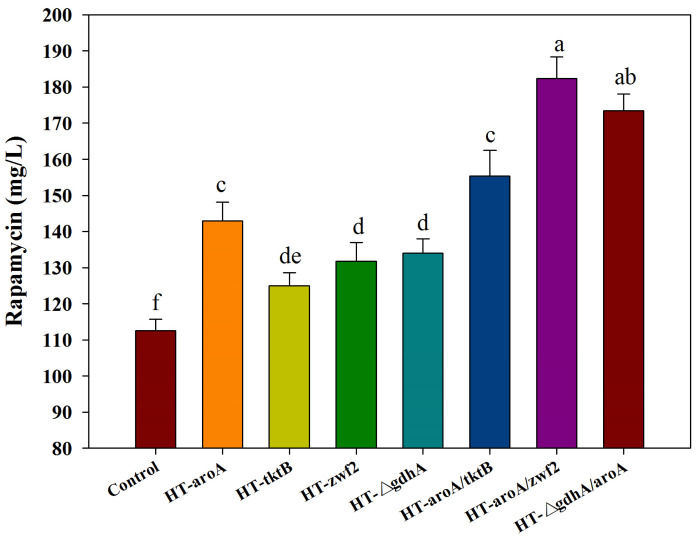
The effect of the single (or multiple) gene manipulation on rapamycin production. *aro*A: 2-dehydro-3-deoxyphosphoheptonate aldolase; *tkt*B: transketolase; *zwf*2: glucose-6-phosphate 1-dehydrogenase; *gdh*A: glutamate dehydrogenase (NADP+). The significant differences (*p* < 0.05) of rapamycin yields were marked with the lowercase letters among the different strains.

According to the analysis of all of the predicted targets, the first four targets (i.e., r47, r51, r279, r532) with the highest *f_PH_* values clearly indicated that the inadequate supply of DHCHC and NADPH might be the key limiting steps in rapamycin biosynthesis. Therefore, the reinforcement of DHCHC and NADPH supply were first selected to verify their effects on rapamycin production by single or multiple gene manipulation. Among the single gene manipulation, the final rapamycin yields showed the significant improvement, to some extent, in all of the engineered strains compared with the control (112.59 mg/L) (Figure 7). Furthermore, the highest yields of rapamycin were observed in HT-aroA (142.99 mg/L), followed by the HT-△gdhA strain (133.98 mg/L), HT-zwf2 (131.73 mg/L), and HT-tktB (124.97 mg/L). These results further confirm that the enhancement of the shikimate pathway and NADPH supply were effective methods in rapamycin overproduction. However, the relatively higher contents of rapamycin in the HT-aroA strain also indicate that the insufficiency of the starting unit (DHCHC) might be a key limiting factor in rapamycin biosynthesis. It is the potential reason for the relatively higher yields of rapamycin which were observed in the HT-aroA strain by the direct enhancement of the shikimate pathway. During multiple gene manipulation, the final rapamycin production increased by approximately 1.38-, 1.62- and 1.54-fold in the engineered strains of HT-aroA/tktB (155.37 mg/L), HT-aroA/zwf2 (182.40 mg/L), and HT-△gdhA/aroA (173.39 mg/L) compared to the wild strain. Notably, the enzymatic reactions catalyzed by the genes of *aro*A and *zwf*2 were all significant relative to erythrose-4-phosphate. Therefore, the highest yields of rapamycin in the HT-aroA/zwf2 strain further confirmed that the inadequate sufficient of DHCHC in the intracellular was the key limiting step for rapamycin overproduction. To sum up, this information not only suggests that the intracellular metabolic flux was clearly disturbed but also indicates that an increase in the NADPH and DHCHC levels was an effective strategy in rapamycin overproduction.

## 4. Discussion

As a type of secondary metabolite synthesized by multiple precursors, the enhancement of its yield is seriously restricted by the complex intracellular metabolic regulatory system. Nowadays, the classical strategies of secondary metabolite overproduction are to strengthen the supply of the precursors and the gene cluster expression of secondary metabolism. However, the large-scale improvement of secondary metabolite yields (e.g., rapmaycin, tacrolimus, ascomycin) is still seriously restricted. An important reason is the insufficient understanding of the distribution and transformation in the intracellular metabolic fluxes during the processing of secondary metabolite biosynthesis. Therefore, in addition to the gene cluster of secondary metabolites, more attention on the global and pleiotropic regulators should be enhanced during the intracellular metabolism. Nevertheless, most of these regulators are often cryptic or exist with a silent/poor expression state under ordinary laboratory fermentation conditions. Therefore, how to wake and induce (or enhance) the expression of these cryptic or silent regulators plays a key role in the further improvement of secondary metabolite yields. In previous reports, chemical elicitors were applied to find novel antibiotics, activating the expression of silent or poorly expressed gene clusters and improving the biochemical product accumulation [18,37]. Therefore, chemical elicitors should not only be recognized as a convenient method to activate these potential regulators but also provide an effective strategy to explore the dynamic changes of intracellular metabolism. Nowadays, some different types of chemical elicitors have been investigated in various secondary metabolite production [16,17,31,32,37]. For example, the biosynthesis of several antibiotics (e.g., actinorhodin, tacrolimus, thiostrepton, streptomycin) can be effectively activated by some specific chemical elicitors, including histone deacetylase (HDAC) inhibitors, signaling molecules, rare earth elements, and sulfo-compounds. However, based on the results of pretesting, the final yields of rapamycin did not exhibit significant differences between the control and the signaling molecule treatments (e.g., *r*-butyrolactones and N-acetylglucosamine). Therefore, SB (HDAC inhibitors), LaCl_3_ (rare earth elements), and DMSO (sulfo-compounds) were finally selected to investigate their effects on the intracellular metabolism during rapamycin accumulation.

The main goal of this work was to perturb the intracellular metabolism using different types of chemical elicitors, explore the rearrangement and transformation of the intracellular metabolic fluxes, and search the potential key limiting steps in rapamycin overproduction. According to the current results, the final rapamycin yields showed obvious improvements after all of the chemical elicitor treatments. The PCA score plots of the intracellular metabolomic datasets also suggested that the intracellular metabolism exhibited the significant differences in each chemical elicitor treatment. Meanwhile, several potential biomarkers (e.g., glyceraldehyde 3-phosphate, fructose 6-phosphate, glycerate 3-phosphate) were identified, most of which displayed a close relationship with the precursor biosynthesis of rapamycin (Figure 3). Notably, the different functional mechanism was observed in rapamycin overproduction in each chemical elicitor treatment by combining it with WGCNA and MetPA analyses (Figure 4 and Figure 5). For the DMSO treatment, while no significantly correlated metabolic pathways were enriched in the precursor biosynthesis of rapamycin, several kinds of fatty acids and stress response factors were identified and showed a stronger correlation with the changes in cell membrane permeability. In fact, fatty acids are not only important components of the cell membrane but also have a significant correlation with the biosynthesis of CoA compounds, such as acetyl-CoA and malonyl-CoA. It is well known that several kinds of CoA compounds are needed in rapamycin biosynthesis, including malonyl-CoA and methylmalonyl-CoA. Therefore, the relatively high abundances of these fatty acids (i.e., dodecanoic acid, decanedioic acid, octadecanoic acid) might also indicate that fatty acids metabolism was activated under the DMSO treatment. The synthesis of these CoA compounds in rapamycin production would be beneficial. Additionally, DMSO is often used as the classical carrier of drugs to cross the cell membrane. According to previous reports, it was found that some secondary metabolite production can be improved by increasing the cellular permeability with DMSO, such as blue pigment [38] and rosmarinic acid [39]. It is worth noting that the highest rapamycin production was observed after feeding DMSO at 72 h of the fermentation (Figure 2). Based on the profiling of the rapamycin accumulation, this time point was the starting point of the rapamycin mass biosynthesis (Figure S2). Therefore, the increase in the cell membrane permeability would be beneficial for the transport of rapamycin from the intracellular to the extracellular, which also weakens the negative influence of rapamycin for normal cell growth.

Different to DMSO, amino acid metabolism was significantly enriched in both of the SB and LaCl_3_ treatments. In fact, amino acid metabolism not only plays important roles in maintaining the normal cellular metabolic function but also provides the crucial precursors (e.g., pipecolic acid) in rapamycin biosynthesis. However, slight differences were also observed. For example, the pyruvate family (valine, leucine, isoleucine, glycine, serine, and threonine) and aromatic amino acids (phenylalanine, tyrosine, and tryptophan) were significantly correlated with the SB and LaCl_3_ treatments, respectively. At the same time, the PP pathway was also enriched in the LaCl_3_ treatments. In the intracellular metabolism, the PP pathway was the main sources of NADPH and provided a key precursor (erythrose 4-phosphate, reaction with PEP) to participate in the shikimate pathway. Therefore, the above obtained information clearly suggested that the key metabolic nodes regulated by these two chemical elicitors were different in the intracellular metabolism, such as PEP for LaCl_3_ and pyruvate for SB. In the previous analysis, the important roles of these two key metabolic nodes was deeply discussed, as both controlled the metabolic fluxes within several precursor synthetic pathways of rapamycin. However, from the perspective of rapamycin formation, the regulation in intracellular metabolic fluxes existed in an obvious chronological order in the SB and LaCl_3_ treatments. Nowadays, it is clear that the formation of DHCHC from chorismic acid (the shikimate pathway) is the first step in rapamycin biosynthesis, which is further extended with malonyl-CoA or methylmalonyl-CoA by multiple successive polyketide chain elongation cycles. Notably, a total of seven methylmalonyl-CoA was required in rapamycin biosynthesis [8], which is larger than other precursors (e.g., DHCHC, pipecolate). Therefore, it is easy to understand that the regulation of an insufficient supply of methylmalonyl-CoA might play an important role in rapamycin overproduction during the SB treatment. However, for the LaCl_3_ treatment, the more important function is to regulate the start of rapamycin biosynthesis.

Based on the above analysis, the current work further identified several potential key limiting targets within the enriched metabolic pathways using the GSMM model (Figure 6). After the corresponding gene manipulation, the obvious improvement in rapamycin production in the engineered strains also further confirmed the potential regulation of intracellular metabolism under these chemical elicitor treatments (Figure 7). These findings not only provide a new insight into a better understanding of the intracellular metabolism of rapamycin overproduction but may also be applied to improve other biochemical product yields.

## Figures and Tables

**Table 1:** Information of all of the distinct metabolic modules identified by WGCNA analysis.

Module	Association	*p* Value	Metabolites
**M1**	*r* = 0.79	*p* = 3 × 10^−4^	Trehalose, dodecanoic acid, decanedioic acid, glucose, glyoxylic acid, glycerol, fructose 6-phosphate, arabinal, 1,3-butanediol, octadecanoic acid, putrescine
**M2**	*r* = 0.69	*p* = 0.005	Methylmalonate, nonanoic acid, niacinamide, 6-phospho-d-gluconate, docosanoic acid, hexadecanoic acid, fructose 1,6-bisphosphate, nonanedioic acid, nicotinate, tetradecanoic acid, glucose 6-phosphate, malonic acid, 2-oxobutanoate
**M3**	*r* = 0.66	*p* = 0.005	Allothreonine, phenylalanine, tyrosine, aspartic acid, threonine, lactate, isoleucine, cadaverine, norleucine, acetoin, butanal
**M4**	*r* = 0.81	*p* = 1 × 10^−4^	Proline, shikimate, glucitol, lysine, indole, xylulose 5-phosphate, tryptophan, rhamnose, picolinic acid, pentanoic acid, linoleic acid, ribulose 5-phosphate, sedoheptulose 7-phosphate, ethanol, erythrose 4-phosphate, ethanedioic acid, alanine

**Table 2:** Information of the potential limiting targets predicted using GSMM model in *S. hygroscopicus*.

Reaction ID	Enzyme	Strategy	Reaction Definition
r532	2-dehydro-3-deoxyphosphoheptonate aldolase	Overexpression	Phosphoenolpyruvate + D-erythrose-4-phosphate + H_2_O → 3-deoxy-D-arabino-heptulosonate-7-phosphate + phosphate
r279	Glutamate dehydrogenase (NADP+)	Knockout	NH_3_ + 2-oxoglutarate + NADPH + H → glutamate + NADP + H_2_O
r47	Transketolase	Overexpression	Erythrose-4-phosphate + xylulose-5-phosphate → fructose-6-phosphate + glyceraldehyde-3-phosphate
r51	Glucose-6-phosphate 1-dehydrogenase	Overexpression	D-glucose-6-phosphate + NADP → D-6-phosphogluconolactone + NADPH + H
r381	Glutaryl-CoA dehydrogenase	Knockout	Glutaryl-CoA + (2) NAD → crotonyl-CoA + (2) NADH + CO_2_
r274	Aspartate aminotransferase	Overexpression	Oxaloacetate + L-glutamate → L-aspartate + alpha-ketoglutarate
r18	Phosphoenolpyruvate carboxylase	Knockout	Phosphoenolpyruvate + CO_2_ + H_2_O → phosphate + oxaloacetate
r23	Pyruvate carboxylase	Knockout	HCO_3_ + pyruvate + ATP → phosphate + oxaloacetate + ADP
r293	Dihydrodipicolinate synthase	Overexpression	Pyruvate + L-aspartate-semialdehyde → (2) H_2_O + L-2,3-dihydrodipicolinate
r421	Aspartate kinase	Overexpression	Aspartate + ATP → aspartyl-4-phosphate + ADP

## Data Availability

The data presented in this study are available on request from the corresponding author.

## Supplementary Materials

The following are available online at https://www.mdpi.com/article/10.3390/microorganisms10112205/s1, Table S1: Strains and plasmids used in this study [40,41,42,43]. Table S2: Primers used in this work. Table S3: List of identified and putatively annotated metabolites measured by GC-MS and LC-MS/MS. Table S4: Optimized multiple reaction monitoring (MRM) parameters for each metabolite by LC-MS/MS analysis. Figure S1: Pathway enrichment analysis of the identified metabolites in each distinct metabolic module by WGCNA analysis under sodium butyrate (A) and LaCl_3_ (B) treatments, respectively. Figure S2: Dynamic fermentation profiles of biomass, residual sugar (glucose form), inorganic ammonium (NH_4_^+^ form), inorganic phosphorus (PO_4_^3+^ form), and rapamycin production in *S. hygroscopicus*.
