# Supplementary material for: Integrated Metabolomic and Network Analysis to Explore the Potential Mechanism of Three Chemical Elicitors in Rapamycin Overproduction
Source: Microorganisms. 2022 Nov 8;10(11):2205. doi: 10.3390/microorganisms10112205 (PMC9698630; doi:10.3390/microorganisms10112205)
Supplement: Supplementary file 1 [file microorganisms-10-02205-s001.zip › microorganisms-1998063-supplementary.pdf]

Supplementary materials:

Integrated metabolomics and network analysis to explore the potential mechanism of three chemical elicitors in rapamycin overproduction

Dandan Zhang, Jinyu Chen, Huizi Wang, Cheng Wang *

College of Forestry, Northwest A&F University, Yangling, Shaanxi, 712100, PR China

*Correspondence to: Cheng Wang

E-mail: jmcookie@nwafu.edu.cn

Phone/Tax: +86 029-87082216

Table S1. Strains and plasmids used in this study.

**Strains/Plasmids**	**Descriptions** [1]	**Source/reference**
**Strains**		
S. hygroscopicus HA12	Parent strain	Derived from *S. hygroscopicus* ATCC 29253
E. coli DH5α	Host for general cloning	Our Lab
Escherichia coli JM109	Plasmid construction and general cloning	TransGen Biotech (Beijing, China)
ET12567/pUZ8002	Non-methylating ET12567 containing non-transmissible RP4 derivative plasmid; pUZ8002, Cm^R, Kan^R	Kieser et al. 2000
HT-*tktB*	*S. hygroscopicus* ATCC 29253 transformed with pTKTB	This study
HT-*aroA*	*S. hygroscopicus* ATCC 29253 transformed with pAROA	This study
HT-*zwf2*	*S. hygroscopicus* ATCC 29253 transformed with pZWF2	This study
HT-△*gdhA*	*S. hygroscopicus* ATCC 29253 with an in-frame deletion of *gdh*A	This study
HT-*aroA*/*tktB*	*S. hygroscopicus* ATCC 29253 transformed with pAT	This study
HT-*aroA*/*zwf2*	*S. hygroscopicus* ATCC 29253 transformed with pAZ	This study
HT-△*gdhA*-*aroA*	HT-△*gdhA* transformed with pAROA	This study
**Plasmids**		
pUC18	*E. coli* cloning vector; Amp^R	Laboratory stock
pIB139	Intergrative plasmid containing *oriT*, *attP*, *int*, *aac(3)IV* and *ermEp**	Wilkinson et al. 2002
pUC119-Kan^R	pUC119 with Kan^R	Xiang et al. 2009
pKC1139	Temperature-sensitive *E. coli*-*Streptomyces* shuttle vector containing *oriT*, aac(3) IV for gene disruption	Bierman et al. 1992
pTKTB	pIB139 based integrative plasmid containing *tktB*, Apr^R	This study
pAROA	pIB139 based integrative plasmid containing *aroA*, Apr^R	This study
pZWF2	pIB139 based integrative plasmid containing *zwf*2, Apr^R	This study
pAZ	pIB139 based integrative plasmid containing *aroA* and *zwf*2, Apr^R	This study
pAT	pIB139 based integrative plasmid containing *aroA* and *tktB*, Apr^R	This study
p△GCDH	pKC1139 based deletion plasmid with in-frame deletion of 750-bp internal to *gdh*A, Apr^R, Kan^R	This study

[1] Cm^R, chloramphenicol resistance; Kan^R, kanamycin resistance; Apr^R, aparamycin resistance; Amp^R, ampicillin resistance; *attP*, plasmid ΦC31 attachment site; *int*, integrase gene, confer the plasmid to integrate in chromosome; *aac(3)IV*, apramycin resistance gene; *oriT*, origin of transfer; *ermEp**, promoter region of the erythromycin resistance gene.

References

Kieser T, Bibb MJ, Buttner MJ, Chater KF, Hopwood DA (2000) Practical *Streptomyces* Genetics. United Kingdom: John Innes Foundation.

Wilkinson CJ, Hughes-Thomas ZA, Martin CJ, Bohm I, Mironenko T, Deacon M, Wheatcroft M, Wirtz G, Staunton J, Leadlay PF (2002) Increasing the efficiency of heterologous promoters in actinomycetes. J Mol Microbiol Biotechnol 4: 417-426.

Xiang SH, Li J, Yin H, Zheng JT, Yang X, Wang HB, Luo JL, Bai H, Yang KQ (2009) Application of a double-reporter-guided mutant selection method to improve clavulanic acid production in *Streptomyces clavuligerus*. Metab Eng 11: 310-318.

Bierman M, Logan R, O'Brien K, Seno ET, Nagaraja RR, Schoner BE (1992) Plasmid cloning vectors for the conjugal transfer of DNA from *Escherichia coli* to *Streptomyces* spp. Gene 116: 43-49.

Table S2. Primers used in this work.

Primer name	Sequence [3] 5'→3'
aroA-F[1]	ATAT**CATATG**TGCCTTCTTTACCGACCTCTG **(NdeI)**
aroA-R[1]	CTTG**TCTAGA**GCGGTCACCCCTGTCATTC **(XbaI)**
aroA-F[2]	ATAT**CATATGT**GCCTTCTTTACCGACCTCTG **(NdeI)**
aroA-R[2]	CTTG**AAGCTT**GCGGTCACCCCTGTCATTC **(HindIII)**
tktB-F[1]	ATAT**CATATG**TGGGTGGAACTCGAAAAGGC **(NdeI)**
tktB-R[1]	CTTG**TCTAGA**GACGGGTTGGTGGTGACGC **(XbaI)**
tktB-F[2]	CTTG**AAGCTT**TGGGTGGAACTCGAAAAGGC **(HindIII)**
tktB-R[2]	CTTG**TCTAGA**GACGGGTTGGTGGTGACGC **(XbaI)**
zwf2-F[1]	ATAT**CATATG**GGAGGCGTGAAACCGTGAG **(NdeI)**
zwf2-R[1]	CTTG**TCTAGA**TGGACCAGAGCGGAGTTGAT **(XbaI)**
zwf2-F[2]	CTTG**AAGCTT**GGAGGCGTGAAACCGTGAG **(HindIII)**
zwf2-R[2]	CTTG**TCTAGA**TGGACCAGAGCGGAGTTGAT **(XbaI)**
gdhA-LF	ATAT**AAGCTT**GAACGGAGCGGTGTATGACG **(HindIII)**
gdhA-LR	CTTG**TCTAGA**GCCACGGTTCACCTGGATGT **(XbaI)**
gdhA-RF	ATAT**GGTACC**CGGTCGCCCTGGTCAAGAA **(KpnI)**
gdhA-RR	ATAT**GAATTC**CCGAAGACCAAGCCGAAGC **(EcoRI)**
pIB-F	TTGCGCCCGATGCTAGTCG
pIB-R	GCACGACAGGTTTCCCGACTG

[1] Primers used in the single gene manipulation;

[2] Primers used in the plasmid construction of two genes co-overexpression (pAT, pAZ).

[3] Underline stand for the restriction sites;

Table S3. List of identified and putatively annotated metabolites measured by GC-MS and LC-MS/MS.

Detection method	Compound	Class
GC-MS	1,3-Butanediol	Other compound
	2-Oxobutanoate	Organic acids
	2-Oxoglutarate	Organic acids
	6-Phospho-D-gluconate	Sugar
	Acetoin	Other compound
	Acetone	Other compound
	Adenosine	Nucleotides
	Alanine	Amino acid & Amides
	Allothreonine	Amino acid & Amides
	Arabinal	Other compound
	Aspartic acid	Amino acid & Amides
	Benzoic acid	Organic acids
	Butanal	Other compound
	Cadaverine	Amino acid & Amides
	Citrate	Organic acids
	Decanedioic acid	Organic acids
	Docosanoic acid	Fatty acids
	Dodecanoic acid	Fatty acids
	Eicosanoic acid	Fatty acids
	Ethanedioic acid	Organic acids
	Ethanol	Other compound
	Fumarate	Organic acids
	Galactose	Sugar
	Glucitol	Other compound
	Glucose	Sugar
	Glucuronic acid	Organic acids
	Glutamic acid	Amino acid & Amides
	Glycerate 3-phosphate	Organic acids
	Glycerol	Fatty acids
	Glycerol 3-phosphate	Fatty acids
	Glycine	Amino acid & Amides
	Glyoxylic acid	Organic acids
	Guanine	Nucleotides
	Guanosine	Nucleotides
	Heptadecanoic acid	Fatty acids
	Hexadecanoic acid	Fatty acids
	Homocystine	Amino acid & Amides
	Homoserine	Amino acid & Amides
	Hydroxylamine	Amino acid & Amides
	Indole	Other compound
	Isoleucine	Amino acid & Amides

Detection method	Compound	Class
GC-MS	Lactate	Organic acids
	Leucine	Amino acid & Amides
	Linoleic acid	Fatty acids
	Lysine	Amino acid & Amides
	Malate	Organic acids
	Malonic acid	Organic acids
	Maltose	Sugar
	Methionine	Amino acid & Amides
	Methylmalonate	Organic acids
	Myo-Inositol	Other compound
	Niacinamide	Amino acid & Amides
	Nicotinate	Organic acids
	Nonadecanoic acid	Fatty acids
	Nonanedioic acid	Organic acids
	Nonanoic acid	Organic acids
	Norleucine	Amino acid & Amides
	n-Pentadecanoic acid	Fatty acids
	Octadecanoic acid	Fatty acids
	Oleic acid	Fatty acids
	Ornithine	Amino acid & Amides
	Oxaloacetate	Organic acids
	Pentanoic acid	Organic acids
	Phenylalanine	Amino acid & Amides
	Picolinic acid	Nucleotides
	Pipecolate	Organic acids
	Proline	Amino acid & Amides
	Putrescine	Amino acid & Amides
	Pyruvate	Organic acids
	Rhamnose	Sugar
	Serine	Amino acid & Amides
	Shikimate	Organic acids
	Succinate	Organic acids
	Tetradecanoic acid	Fatty acids
	Threonine	Amino acid & Amides
	Thymidine	Nucleotides
	Trehalose	Sugar
	Tryptophan	Amino acid & Amides
	Tryptophol	Other compound
	Tyrosine	Amino acid & Amides
	Uracil	Nucleotides
	Urea	Amino acid & Amides
	Valine	Amino acid & Amides

**Detection method**	**Compound**	**Class**
LC-MS/MS	Erythrose 4-phosphate	Sugar
	Fructose 6-phosphate	Sugar
	Fructose 1,6-bisphosphate	Sugar
	Glucose 6-phosphate	Sugar
	Glyceraldehyde 3-phosphate	Other compound
	Phosphoenolpyruvate	Organic acids
	Ribulose 5-phosphate	Sugar
	Ribose 5-phosphate	Sugar
	Sedoheptulose 7-phosphate	Sugar
	Xylulose 5-phosphate	Sugar

Table S4. Optimized multiple reaction monitoring (MRM) parameters for each metabolite by LC-MS/MS analysis.

Compound	Molecular weight	Precursor ion m/z	Product ion m/z	Product formula	De-clustering potential (V)	Collision energy (V)
Glucose 6-phosphate	260.14	259.1	97.0	$[H_2PO_4]^-$	-65	-20
Fructose 1,6-bisphosphate	340.12	339.1	97.0	$[H_2PO_4]^-$	-97	-26
Fructose 6-phosphate	260.14	259.1	97.0	$[H_2PO_4]^-$	-65	-18
Glyceraldehyde 3-phosphate	170.06	169.0	97.0	$[H_2PO_4]^-$	-48	-10
Phosphoenolpyruvate	168.04	167.0	78.9	$[PO_3]^-$	-50	-16
Ribulose 5-phosphate	230.11	229.1	97.0	$[H_2PO_4]^-$	-35	-13
Ribose 5-phosphate	230.11	229.1	97.0	$[H_2PO_4]^-$	-35	-13
Xylulose 5-phosphate	230.11	229.1	97.0	$[H_2PO_4]^-$	-100	-12
Sedoheptulose 7-phosphate	290.16	289.1	97.0	$[H_2PO_4]^-$	-33	-22
Erythrose 4-phosphate	200.08	199.1	97.0	$[H_2PO_4]^-$	-40	-17

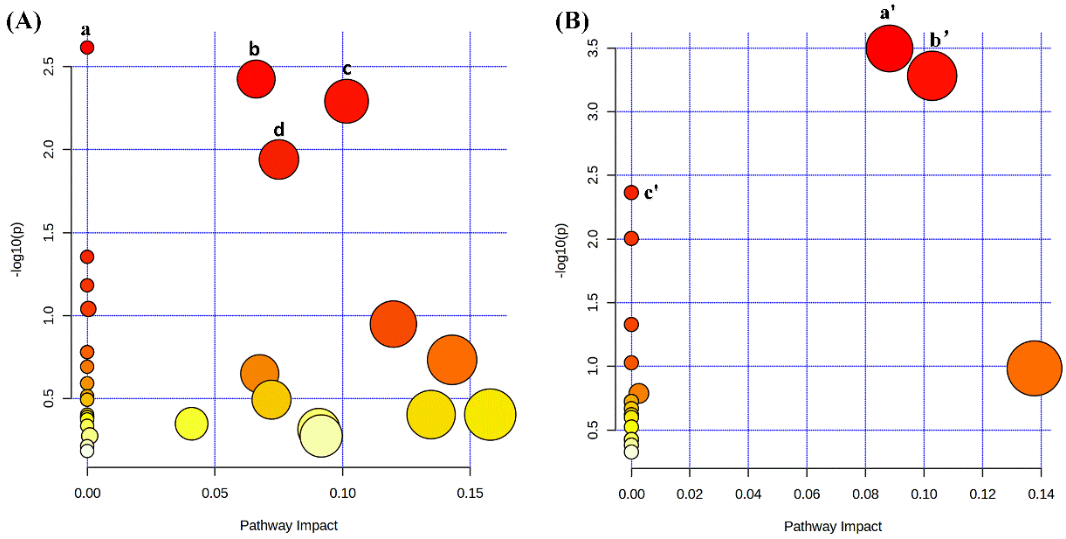

Fig. S1 Pathway enrichment analysis of the identified metabolites in each distinct metabolic module by WGCNA analysis under sodium butyrate (**A**) and $LaCl_3$ (**B**) treatments, respectively. The screening parameter is *p* value < 0.05 and metabolites number ≥ 3. Three chemical elicitors were simultaneously added into the medium at 72 h of the fermentation (i.e., DMSO, $LaCl_3$, Sodium butyrate (SB)). The metabolomics dataset was derived from the samples collected at 24 h after feedings chemical elicitors. In plot of **(A)**: **a**: Aminoacyl-tRNA biosynthesis; **b**: Nicotinate and nicotinamide metabolism; **c**: Glycine, serine and threonine metabolism; **d**: Valine, leucine and isoleucine biosynthesis. In plot of **(B)**: **a'**: Phenylalanine, tyrosine and tryptophan biosynthesis; **b'**: Pentose phosphate pathway; **c'**: Aminoacyl-tRNA biosynthesis.

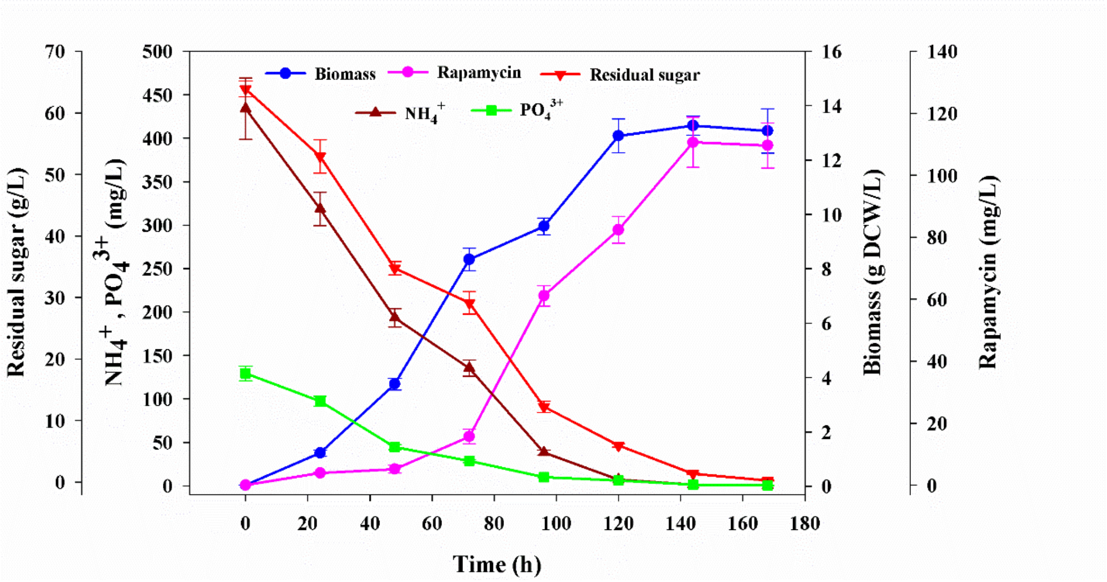

Fig. S2 Dynamic fermentation profiles of biomass, residual sugar (glucose-form), inorganic ammonium (NH_4^+-form), inorganic phosphorus (PO_4^{3+}-form) and rapamycin production in *S. hygroscopicus*.
